# Striving Towards National Lower-Risk Gambling Guidelines: An Empirical Investigation Among a Sample of Swedish Gamblers

**DOI:** 10.1007/s10899-024-10372-w

**Published:** 2025-01-08

**Authors:** Nicki A. Dowling, Peter Wennberg, Håkan Wall, Olof Molander

**Affiliations:** 1 Department of Clinical Neuroscience, Centre for Psychiatry Research, Karolinska Institutet, Solna, Sweden; 2 Department of Public Health Sciences, Stockholm University, Stockholm, Sweden; 3 Department of Global Public Health, Karolinska Institutet, Solna, Sweden; 4 Department of Psychology, Inland Norway University of Applied Sciences, Lillehammer, Norway; 5 School of Psychology, Deakin University, Burwood, Australia; 6 Centrum för psykiatriforskning, Norra Stationsgatan 69, plan 7, Stockholm, 113 64 Sweden

**Keywords:** Gambling consumption, Lower-risk limits, Guidelines, Harms, Prevention

## Abstract

Several countries, including Canada and Australia, have developed public health-based lower-risk gambling limits to differentiate lower-risk from higher-risk gambling. This study aimed to identify a preliminary set of lower-risk gambling limits (gambling frequency, duration, expenditure, expenditure as a proportion of personal net income, and diversity), and investigate if gambling types are linked to additional harms, in a Swedish context. The study involved secondary analyses of two online survey studies using the Gambling Disorder Identification Test (GDIT). Receiver operating curve analyses were conducted in relation to both + 1 and + 2 gambling-related harms in a sample of 705 past-year gamblers. Potential lower-risk limits ranges identified were: gambling frequency of “2–3 times a week” to “4 or more times a week” (8–16 times monthly); gambling duration of 6 to 15 h per month; gambling expenditure of 2,000 SEK (approximately $USD190) per month; gambling expenditure as a proportion of personal net income of 5%; and gambling diversity of only one problematic gambling type. Gambling on slots and sports betting were associated with gambling-related harms. The lower-risk limits in the current study were higher than in previous studies, which may be explained by the large proportion of support- or treatment-seeking gamblers with high rates of problem gambling and problematic online gambling in the study sample. An international consensus-based framework on gambling consumption is warranted, with lower-risk limits validated in future empirical studies using larger datasets collected from the Swedish general population.

**Supplementary Information:**

The online version contains supplementary material available at 10.1007/s10899-024-10372-w.

Gambling is a major public health concern globally (Ukhova et al., 2024). The prevention of Gambling Disorder (GD) (American Psychiatric Association, 2013) is informed by public health perspectives, which aim to identify its determinants and subsequent harms (Korn & Shaffer, 1999). There is evidence that gambling-related harms, such as financial loss, relationship breakdown, and psychological distress (Langham et al., 2016), can occur below the GD diagnostic thresholds (Browne & Rockloff, 2017; Delfabbro & King, 2019), thereby providing the opportunity for public health initiatives to impact on a larger segment of the population (Currie et al., 2006, 2009). One such initiative to reduce alcohol-related harm has been the adoption of lower-risk drinking guidelines (Room & Rehm, 2012; Wechsler et al., 1995), which are promoted to the general public and clinical populations to help individuals adapt safe behaviors in response to accurate information (Room & Rehm, 2012).

## The Identification of Lower-Risk Gambling Limits

An emerging literature for gambling lower-risk limits in population-representative studies conducted in Canada (Currie et al., 2006, 2008, 2009, 2012, 2017), Germany (Brosowski et al., 2015), Australia (Dowling et al., 2021; Dowling et al., 2021), and Norway (Langeland et al., 2022) have identified relatively consistent limits across multiple gambling consumption indices: gambling frequency (0.6–5 times per month or 2–4 activities per year); gambling duration (400–454 min per year); gambling expenditure (USD$93–$720 per year); and gambling expenditure as a proportion of income (1–3% of gross household income/0.8–10% of gross personal income). More recently, lower-risk limits were identified via linked analyses with 11 high-quality population-level datasets across eight Western countries: gambling no more than 1% of household income, gambling no more than 4 days per month, and avoid regularly gambling at more than 2 types of games (i.e., gambling diversity, number of gambling types) (see https://gamblingguidelines.ca/*)* (Hodgins et al., 2023). Other studies extending this research to specific groups, including post-treatment-seeking gamblers (Weinstock et al., 2007), university students (Weinstock et al., 2008), and community/psychiatric outpatients (Quilty et al., 2014) have identified similar lower-risk limits. In contrast, studies using behavioral data from gambling website customers have identified limits that are higher than those derived from prevalence study data (Jonsson et al., 2022; Louderback et al., 2021).

These studies, which suggest that lower-risk gambling limits generally yield moderate levels of classification accuracy, have most often assigned equal importance to both sensitivity (accurate identification of people experiencing harm) and specificity (accurate identification of people not experiencing harm). There are some exceptions, however, whereby some studies have conducted analyses that maximize specificity, with a view to increasing the credibility of the limits by reducing the proportion of false positives (Currie et al., 2017; Dowling et al., 2021a; Hodgins et al., 2023), while others have conducted analyses that maximize sensitivity, arguing that the consequences of setting thresholds too high are more serious than the consequences of setting thresholds too low, consistent with public health’s ”precautionary principle” (Dowling et al., 2021). The extant research has also defined gambling-related harm using diagnostic criteria or subsets of items from measures of problem gambling severity, with more recent research employing validated measures of gambling-related harm (Dowling et al., 2021). Limits are generally robust to these definitional variations in gambling-related harm, although superior parameters are generally produced when gambling-related harm is defined as two or more harms (Currie et al., 2006, 2008, 2009; Dowling et al., 2021a).

## Harmful Gambling Types

While most of the existing evidence base has identified lower-risk limits applicable across all gambling activities, research has suggested that there are considerable variations in risk associated with specific types of gambling. Various studies indicate that certain gambling activities pose higher risk and associated harm (Binde et al., 2017; Browne et al., 2023; Currie et al., 2006, 2009; Delfabbro et al., 2020; Dowling et al., 2005; Markham et al., 2016; Russell et al., 2023; Wall et al., 2021). Specifically, these studies have found that electronic gaming machines (EGMs or slots) contribute more to the development of gambling problems and harm than other gambling activities, mainly because of their structural characteristics, such as rapid playing speeds and payout intervals, use of multi-credit or -lines, note acceptors, and credited wins. They also reveal that casino table games, sports betting, and racing are high-risk activities relative to other forms of gambling.

### Study Aims

To date, no studies have specifically attempted to derive lower-risk gambling limits in a Swedish context. The aim of this study was therefore to identify a preliminary set of lower-risk limits for gambling consumption indices in Sweden. A secondary aim was to investigate which problematic gambling types were associated with harm beyond the effect of the gambling consumption indices.

## Methods

### Participants and Study Design

This study secondarily analyzed data from two previous online survey studies conducted in Sweden that included the Gambling Disorder Identification Test (GDIT) (Molander et al., 2021). The first study involved gamblers recruited from treatment- and helpline support-seeking contexts, self-help groups, and social media (Molander et al., 2021). Inclusion criteria included: ≥18 years old and past-year gambling participation (excluding the self-help group participants). The second study involved support-seeking gamblers recruited via advertisements on the Swedish gambling helpline (Wall et al., 2025). Inclusion criteria included: a desire for support for their own gambling and visiting the helpline’s website. Both studies received approval from the Regional Ethics Board of Stockholm, Sweden (ref. no. 2017/1479-31 and 2022-03651-01).

The combined sample comprised 705 past-year gamblers from: treatment services (*n* = 78, 11.1%), helpline support services (*n* = 291, 41.3%), self-help groups (*n* = 47, 6.7%), and social media (*n* = 289, 41.0%). As indicated in Table 1, GDIT scores were classified as 45.5% for recreational/at-risk gambling, 6.8% for problem gambling, and 47.7% for GD (6.7% mild, 6.7% moderate, 34.3% severe). The mean age of the sample was 33.8 years (SD = 12.6), with 72.6% men. Approximately one-third were university-educated (32.8%) and half were in cohabiting households (52.1%). Two-thirds were employed (66.0%), and the median monthly income was approximately 20,000 Swedish crowns (SEK; USD$1,800). One-quarter of the sample had gambling debts, with a reported median debt of 300,000 SEK.

**Table 1:** Participant characteristics

	Gambling severity^a^					Total *N* = 705 (100%)
	Recreational /at-risk *n* = 321 (45.5%)	Problem gambling *n* = 48 (6.8%)	Gambling Disorder			
			Mild *n* = 47 (6.7%)	Moderate *n* = 47 (6.7%)	Severe *n* = 242 (34.3%)	
Age, M (Sd)	31.4 (12.9)	32.0 (10.6)	35.4 (15.3)	36.2 (11.0)	36.6 (11.7)	33.8 (12.6)
Gender, %						
Men	79.8%	77.1%	78.7%	72.3%	61.2%	72.6%
Women	19.0%	22.9%	19.1%	27.7%	37.6%	26.2%
Can/will not disclose	1.2%	0.0%	2.1%	0.0%	1.2%	1.1%
Highest level of education, %						
University	42.4%	22.9%	29.8%	25.5%	24.0%	32.8%
High school	45.2%	60.4%	57.4%	63.8%	59.5%	53.2%
Junior high school	9.7%	8.3%	10.6%	4.3%	12.8%	10.4%
Civil status, %						
Cohabiting	56.4%	52.1%	40.4%	46.8%	49.6%	52.1%
Children	35.8%	41.7%	42.6%	48.9%	55%	44.1%
Source of income, %						
Employed	61.4%	62.5%	66.0%	59.6%	74.0%	66.0%
Studies	28.0%	20.8%	21.3%	14.9%	6.6%	18.9%
Other^b^	10.6%	16.7%	12.8%	25.5%	19.4%	15.2%
Net income, past month						
SEK, median (IQR), range	18,500 (17,500), 0- 85,000 SEK	20,000 (17,250), 45- 52,000 SEK	23,330 (18,750), 0- 75,000 SEK	21,500 (15,275), 0- 84,500 SEK	21,000 (11,380), 0- 100,000 SEK	20,000 (15,700), 0- 100,000 SEK
Having gambling debts, %	9.0%	27.1%	53.2%	57.4%	80.2%	40.9%
SEK, median (IQR), range	250,000 (595,000), 20 - 8,500, 000 SEK	725,000 (859,000), 300–2,000,000 SEK	225,000 (583,750), 1 - 1500,000 SEK	350,000 (537,500), 100- 1,400,000 SEK	300,000 (640,000), 0- 5,000,000 SEK	300,000 (640,000), 0- 8,500,000 SEK

Note.^a^Gambling severity assessed using the GDIT

^b^This category comprises unemployment insurance, income support, sickness compensation, sickness benefit, pension, and other sources of income

IQR = Interquartile range

SEK = Swedish crowns

### Measures

Participants responded to an online survey, comprising demographic characteristics (age, gender, highest level of education, civil status, source of income, net income, gambling debts). Gambling consumption indices (frequency, duration, expenditure, expenditure as a proportion of income, diversity [i.e., number of problematic gambling types]), gambling harm, and specific problematic gambling types were measured by the GDIT (see Table 2). The GDIT (Molander et al., 2023) consists of 14 items with frequency- and time-based responses across three subscales: Gambling Behavior (items 1–3, scored 0–6), Gambling Symptoms (items 4–10, scored 0–4), and Negative Consequences (items 11–14, scored 0, 2 or 4). The maximum GDIT total score is 62. The GDIT also includes items in an appendix measuring past-month expenditures and gambling types with negative consequences (broken down via internet/online and physical venue). The GDIT is in the public domain, available at www.gditscale.com.

**Table 2:** Gambling consumption indices and harm measurement

Gambling consumption indices	
Gambling frequency	Gambling frequency was assessed using the first item (GDIT_item1_) of the Gambling Behavior subscale: “How often do you gamble?“, with response options of “Never” (0), “Monthly or less” (1), “2–4 times a month” (2), “2–3 times a week” (3), “4 or more times a week” (4), “Daily” (5), “Several times a day” (6).
Gambling duration	Gambling duration was assessed using the gambling frequency item (GDIT_item1_) and the second item (GDIT_item2_) of the Gambling Behavior subscale: “How much time do you spend gambling on a typical day?“, with responses of “No time” (0), “Less than an hour” (1), “1–2 hours” (2), “3–4 hours” (3), “5–6 hours” (4), “7–9 hours” (5), and “10–24 hours” (6). Gambling duration was derived by first recoding GDIT_item1_ and GDIT_item2_ into frequencies per month, i.e., 0, 0.5, 1.5, 3.5, 5, 8 and 17 h, and 0, 1, ,3, 10, 16, 30 and 30 days, respectively. These recoded variables were then multiplied with each other.
Gambling expenditure/gambling expenditure as a proportion of income	Gambling expenditure was assessed using one item in the GDIT appendix “How much money did you wager last month on gambling?” (GDIT_wager item_). The expenditure as a proportion of income was estimated by dividing the appendix items (GDIT_wager item_) by “What was your income after tax last month (including salary and grants)?” (GDIT_income item_), multiplied by 100.
Gambling diversity	Gambling diversity was assessed in a checkbox grid in the GDIT appendix, specifying engagement in problematic gambling activities (Slots, Poker, Casino table games, Sports betting, Horse betting, Bingo, Lotteries, and Instant lotteries/Scratch cards), assessed online or at venues. These items were summarized into binary variables for access mode (any online or venue gambling, regardless of type), or specific activities (regardless of access mode).
Gambling harm	Gambling harm was measured using the Negative Consequences subscale, encompassing past-year gambling-related worsened mental health (GDIT_item12_), experiences of financial problems (GDIT_item11_), or relationship problems (GDIT_item13_), or work or school difficulties (GDIT_item14_). The past-year negative consequences/harms endorsed in GDIT_items11−14_ (excluding responses “Yes, but not in the past year”) were summarized into a numeric variable (1–4 harms). Analyses were then conducted using cut-offs of + 1 harms (to prioritize sensitivity) as well as + 2 harms (in line with most previous research on lower-risk gambling limits.
Problematic gambling types	The checklist in the GDIT appendix used to calculate gambling diversity was employed to measure problematic gambling types. These were dichotomized into a binary variable for each specific gambling type (collapsed online and venue gambling). For descriptive purposes, they were also dichotomized into a binary variable for online or venue gambling (collapsed specific gambling types).

### Statistical Analysis

An analysis plan was published (https://aspredicted.org/vf8mz.pdf), outlining the study’s statistical methods a priori. The final study sample deviated slightly from the plan due to the removal of data from four participants. Monthly incomes over 100,000 SEK (*n* = 14), one reported monthly wager of 150,000,000 SEK, and one reported gambling debt of 40,000,000 SEK were deemed outliers by visual inspection and were replaced with missing values. Regression analyses examining whether problematic gambling activities were associated with harm beyond the effect of the gambling consumption indices, were also subsequently added as a complementary post-hoc analysis.

Descriptive data were presented in mean, standard deviations, and percentages. Numeric variables, including SEK, were presented using median, interquartile range, and range values. Lower-risk gambling limits were identified using Receiver Operating Curve (ROC) analyses across the gambling consumption indices (frequency, duration, expenditure, expenditure as a proportion of income and diversity) in relation to + 1 and + 2 harms. Sensitivity and 1-specificity were plotted for each level of gambling consumption, and the area under the curve (AUC) was estimated for the resulting ROC graph. AUC is a general index of a test’s classification performance, interpreted according to the following guidelines: small (0.50–0.70), moderate (0.70–0.90), and high (> 0.90) classification accuracy (Swets et al., 2000). In the current study, AUC estimates ranged from 0.67 to 0.77, and all values except two demonstrated moderate classification accuracy (i.e., above 0.70). Lower-risk gambling consumption limit ranges were determined based on the highest Youden Index values across + 1 and + 2 harms. The Youden Index provides equal weighting to sensitivity and specificity, thereby minimizing both false positives and false negatives (Ruopp et al., 2008; Youden, 1950).

To examine whether problematic gambling activities were associated with harm beyond the effect of the gambling consumption indices, a stepwise logistic regression approach was employed. First, bivariate associations were estimated between specific gambling types (collapsed online and venue gambling) and + 1/+2 harms, using separate regression models. Second, all gambling types were included in a combined model (Model 1). Finally, the gambling consumption indices were included in a total model (Model 2), where the risk for harms was adjusted for by specific problematic gambling activities. For this full model, participants with a past-month percentage of income wagered larger than 100 were excluded, leaving *n* = 602 (see Table 3). The magnitude of odds ratios (ORs) (and their reciprocal estimates) was interpreted as small (OR = 1.68), medium (OR = 3.47), or large (OR = 6.71) (Fey et al., 2023).

**Table 3:** Association between gambling participation, at-risk indicators, and harms/negative consequences

Predictors	Bivariate associations OR (95% CI), *n* = 705	Model 1 OR (95% CI), *n* = 705	Model 2, full model OR (95% CI), *n* = 602
+ 1 harms			
* Gambling consumption indices*			
Frequency			1.03 (0.94–1.13)
Duration (hours per month)	-	-	1.33 (1.21–1.47)***
Expenditure			1.24 (1.10–1.39)***
Expenditure as a proportion of income	-	-	1.02 (0.92–1.13)
Diversity (number of gambling types)	-	-	1.01 (0.91–1.13)
* Problematic gambling types*			
Slots	7.80 (5.59–10.99)***	8.21 (5.72–11.93)***	1.28 (1.18–1.38)***
Poker	1.65 (1.07–2.54)*	1.07 (0.62–1.85)	0.96 (0.87–1.07)
Casino table games	3.33 (2.31–4.85)***	1.90 (1.21–3.00)**	1.07 (0.97–1.18)
Sports betting	1.65 (1.14–2.42)**	2.31 (1.43–3.72)***	1.11 (1.01–1.21)*
Horse betting	1.48 (0.91–2.41)	1.20 (0.64–2.21)	0.99 (0.88–1.12)
Bingo	1.48 (0.71–3.12)	0.84 (0.32–2.26)	0.96 (0.81–1.13)
Lotteries	1.00 (0.56–1.78)	0.67 (0.26–1.59)	1.02 (0.89–1.19)
Scratch cards	1.30 (0.74–2.27)	0.66 (0.29–1.48)	0.97 (0.83–1.12)
	Model fit	*R*^2^ = 0.32	*R*^2^ = 0.56
+ 2 harms			
*Gambling consumption indices*			
Frequency			1.11 (1.01–1.21)*
Duration (hours per month)			1.18 (1.07–1.3)**
Expenditure			1.17 (1.04–1.31)**
Expenditure as a proportion of income			0.93 (0.83–1.03)
Diversity (number of gambling types)			1.01 (0.9–1.13)
*Problematic gambling types*			
Slots	6.08 (4.25–8.79)***	6.62 (4.48–9.95)***	1.25 (1.16–1.35)***
Poker	1.37 (0.86–2.14)	0.84 (0.47–1.44)	0.96 (0.87–1.06)
Casino table games	2.56 (1.78–3.70)***	1.41 (0.90–2.20)	1.05 (0.95–1.16)
Sports betting	1.91 (1.29–2.81)**	2.61 (1.59–4.30)***	1.16 (1.06–1.27)***
Horse betting	1.65 (1.00–2.71)*	1.15 (0.60–2.16)	0.99 (0.88–1.11)
Bingo	1.65 (0.76–3.46)	0.82 (0.30–2.12)	0.96 (0.81–1.13)
Lotteries	1.4 (0.75–2.52)	0.89 (0.34–2.21)	1.04 (0.9–1.2)
Scratch cards	1.74 (0.98–3.07)	0.99 (0.43–2.22)	1.01 (0.87–1.17)
	Model fit	*R*^2^ = 0.24	*R*^2^ = 0.40

*p < .05, **p < .01, *** *p* < .001

OR = Odds ratios, CI = Confidence intervals, *R*^2^ = Nagelkerkes quasi *R*^2^ (Nagelkerke, 1991)

Model 1 included Slots, Poker Casino table games, Sports betting, Horse betting, Bingo, Lotteries and Scratch cards (regardless of access mode). The full Model 2 included past month expenditure as a proportion of income (dichotomized > 5%), past month duration (dichotomized > 6 h), gambling diversity (dichotomized > 1 gambling types), and Slots, Poker Casino table games, Sports betting, Horse betting, Bingo, Lotteries and Scratch cards (regardless of access mode)

## Results

### Descriptive Statistics

Table 4 shows that the GDIT total score for the sample was 21.1, which is classified in the mild GD category. Participants reported a mean gambling frequency of “2–3 times a week” and a mean past-month gambling duration of 47.5 h (SD = 98.1, range = 0-510). The median past-month gambling expenditure was 600 SEK (approximately USD$60), and the median expenditure as a proportion of income was 5.3%. For gambling diversity, the mean number of gambling types was 1.3. Online gambling was more frequently reported compared to gambling at venues (68.2% versus 26.7%).

**Table 4:** Gambling characteristics (*N* = 705)

	Gambling severity^a^					Total sample
	Recreational /at-risk	Problem gambling	Gambling Disorder			
			Mild	Moderate	Severe	
GDIT total score, M (Sd)	6.4 (3.6)	16.7 (1.4)	21.6 (1.5)	26.9 (1.5)	40.8 (7.9)	21.1 (16.3)
Gambling behavior						
Gambling frequency (GDIT_item1_), mean (frequency)	1.5 (2–4 times a month)	1.9 (2–4 times a month)	2.8 (2–3 times a week)	2.9 (2–3 times a week)	4.2 (4 or more times a week)	2.6 (2–3 times a week)
Gambling duration, hours past month, mean (sd)	3.2 (7.3)	10.2 (14.3)	43.5 (91.7)	35.1 (50.8)	116.9 (134.7)	47.5 (98.1)
Gambling expenditures, past month						
SEK wagered, median (IQR), range	162 (500), 0–150,000 SEK	500 (2,000), 0-534,125 SEK	3,000 (8,900), 0–1,500,000 SEK	5,000 (11,500), 0-200,000 SEK	10,000 (17,625), 0–900,000 SEK	600 (7,970), 0–1,500,000 SEK
Percent of monthly income wagered, median (IQR)	1.1 (4.0)%	5.4 (12.0)%	15.6 (39.6)%	32.4 (59.7)%	50.0 (83.3)%	5.6 (50.0)%
Gambling diversity						
Number of problematic gambling types, mean (sd), range	0.69 (1.17), 0–8 types	1.60 (1.67), 0–8 types	1.45 (1.14), 0–6 types	1.77 (1.56), 0–8 types	1.91 (1.37), 0–8 types	1.29 (1.42), 0–8 types
Problematic gambling types						
Online gambling(%)	40.2%	81.2%	83.0%	87.2%	96.3%	68.2%
Venue gambling (%)	21.8%	20.8%	40.4%	25.5%	31.8%	26.7%
Slots (%)	14.6%	56.2%	53.2%	66.0%	74.4%	44.0%
Poker (%)	8.1%	22.9%	19.1%	17.0%	17.8%	13.8%
Casino games (%)	9.3%	20.8%	34.0%	36.2%	36.4%	22.8%
Sports betting (%)	14.0%	16.7%	19.1%	21.3%	26.0%	19.1%
Horse betting (%)	6.9%	16.7%	10.6%	12.8%	13.6%	10.5%
Bingo (%)	3.4%	4.2%	2.1%	4.3%	5.8%	4.3%
Lotteries (%)	6.9%	14.6%	2.1%	6.4%	7.0%	7.1%
Scratch cards (%)	5.6%	8.3%	4.3%	12.8%	9.9%	7.7%

Note.^a^Gambling severity assessed using the GDIT

### Identification of Lower-Risk Gambling Limits

Lower-risk limits were derived using ROC analyses in relation to both + 1 and + 2 gambling-related harms. A gambling frequency of “2–3 times a week” to “4 or more times a week” (8–16 times monthly) demonstrated sensitivities of 0.54 and 0.71, specificities of 0.75 and 0.87, and Youden’s indices of 0.40 and 0.45 (see supplementary Table 1). Regarding duration, gambling for 6 to 15 h per month demonstrated sensitivities of 0.69 and 0.80, specificities of 0.67 and 0.82 and Youden’s indices of 0.51 and 0.74 (see supplementary Table 2). In terms of expenditures, a past-month wagering of 2,000 SEK showed sensitivities of 0.69 and 0.70, specificities of 0.79 and 0.70, and Youden’s indices of 0.48 and 0.40 (see supplementary Table 3). A ratio of 5% of expenditure as a proportion of income corresponded to sensitivities of 0.74 and 0.71, specificities of 0.69 and 0.58, and Youden’s indices of 0.43 and 0.29 (see supplementary Table 4). For diversity, gambling on one problematic gambling type showed sensitivities of 0.92 and 0.95, specificities of 0.49 and 0.42, and Youden’s indices of 0.41 and 0.37 (see supplementary Table 5).

### Harmful Gambling Types

The gambling consumption indices were included in logistic regression analysis, where the risk for harms was adjusted for participation in specific gambling types (Model 2; see Table 3). The results indicated that two types of gambling—slots and sports betting—were associated with harms. Moreover, the analysis showed that gambling expenditure as a proportion of income and duration during the past month were significant predictors of gambling-related harms (*p* < .001). However, gambling frequency and diversity were not associated with harms (See Table 3).

## Discussion

### Study Findings

This study aimed to identify a preliminary set of lower-risk limits for gambling consumption for Sweden. The results identified limits relating gambling between “2–3 times a week” to “4 or more times a week” (8–16 times per month), between 6 and 15 h per month, 2,000 SEK (approximately USD$190) per month, and 5% of personal net income. These limits are generally higher than those identified in previous research internationally. This is likely explained by the large proportion of treatment- or support-seeking gamblers with high rates of gambling problems in the study sample, given that problem gambling severity and gambling-related harm are overlapping constructs (Browne & Rockloff, 2017; Delfabbro & King, 2019). Relatedly, there was a high proportion of problematic online gamblers in the study sample, whereby problematic online gambling was reported by nearly seven out of 10 participants. Previous studies that have employed behavioral data from online gamblers have identified higher limits than those identified in the general population, possibly because online gambling has a higher risk profile (Jonsson et al., 2022). Although it therefore remains unclear as to how applicable the limits identified in the current study are to the general Swedish population, they offer insights into lower-risk gambling levels in a health-care or support context, which holds merit in relation to clinical applications.

In contrast, the results suggest that engaging in one single problematic gambling type could be related to harm, which is a lower threshold than those identified in previous studies. This finding can be partly explained by the reporting of only problematic gambling types, as recommended in a consensus agreement among gambling researchers (Walker et al., 2006). Further, when investigating engagement in specific problematic gambling types, slots and sports betting were associated with harm. The current study therefore adds to a growing empirical base suggesting that gambling participation in specific types of gambling, such as slots and sports betting, is harmful (Binde et al., 2017; Browne et al., 2023; Currie et al., 2006, 2009; Delfabbro et al., 2020; Dowling et al., 2005; Markham et al., 2016; Russell et al., 2023; Wall et al., 2021), which may have implications for the development of lower-risk limits for specific gambling types (Brosowski et al., 2015; Dowling et al., 2021a; Dowling et al., 2021; Quilty et al., 2014).

### Study Implications

This study was the first step in a larger pursuit to establish national lower-risk gambling guidelines in Sweden. Such guidelines are important to establish for several reasons (Currie et al., 2006, 2008, 2009, 2012, 2017; Quilty et al., 2014; Weinstock et al., 2007, 2008). First, from a public health perspective, lower-risk gambling limits can serve as benchmarks for non-harmful gambling within the general population. Such limits can be used to generate public discussion regarding gambling norms, enable consumers to compare their gambling with these norms, and allow consumers to make informed choices. They can also assist gamblers to reduce their own gambling behavior by increasing their awareness about levels of risk, highlighting potential harms should they exceed the limits, and enhancing motivation to seek help. Second, the establishment of lower-risk gambling guidelines is important from the perspective of the ethical provision of gambling products and industry standards of duty of care. According to the Swedish Gambling Act (Gambling Act 2018 (2018:1138), n.d.), licensed gambling companies are obliged to protect their customers from harms, and subsequently intervene when there is reason to do so. The absence of a uniform set of lower-risk gambling guidelines at a societal level might, from this point of view, result in varying levels of risk employed by different operators and a duty of care that is inconsistently applied. Third, lower-risk limits can be used to monitor the prevalence of gambling-related harm and investigate the impact of secondary intervention efforts. Finally, lower-risk gambling limits are important to establish from a clinical perspective, as such limits can be used as a cost-effective method of screening for people at high-risk of gambling harm in clinical settings and treatment outcome studies and also be applied to gamblers who select non-abstinence treatment goals.

### Study Strengths and Limitations

A strength of the current study is the use of the GDIT instrument to measure gambling-related harm, rather than using subsets of items from problem gambling severity measures. As the first to identify lower-risk gambling limits using this measure, this study adds to the emerging research employing validated measures of gambling-related harm (Dowling et al., 2021). Moreover, by incorporating responses based on frequencies or time (Molander et al., 2024), the GDIT enables a comprehensive and systematic assessment of consumption indices relevant for lower-risk gambling limits. In terms of limitations, the study sample was not drawn from the general population and was over-represented in treatment- and support-seeking gamblers, with high rates of gambling problems and problematic online gambling behavior. The limits identified in this study should therefore be considered preliminary, pending validation by future studies with data collected from large, population-representative samples.

## Conclusion

Lower-risk gambling limits differentiating lower-risk from high-risk gambling behavior, which are identified empirically, can be converted to lower-risk gambling guidelines, which are promoted to the general public. From an authority perspective, public health considerations are important for the establishment of guidelines at a societal level, such as promoting credible thresholds which can be easily remembered. To date, there exists no universal definition of lower-risk gambling and different at-risk indicators, timeframes and currencies have been employed across studies. Consensus-based methods are therefore arguably the next research step in the development of establishing lower-risk gambling guidelines in Sweden. In such studies, Swedish and international gambling researchers, as well as various representatives of Swedish gambling-relevant authorities, can reach consensus on a set of lower-risk gambling principles. Similar research identifying expert and public perspectives regarding the number of limits, the gambling consumption indices, the timeframes employed, and the harms that should be considered, as well as issues relating to the measurement of these constructs and the promotion of such limits have been conducted in other jurisdictions (Currie et al., 2008; Dowling et al., 2021a). This framework can then be validated in future empirical studies, using larger datasets collected from the Swedish general population.

## Electronic Supplementary Material

Below is the link to the electronic supplementary material.

Supplementary Material 1

## Data Availability

Due to the nature of this research, participants of this study did not agree for their data to be shared publicly, so supporting data is not available.

## References

[CR1] American Psychiatric Association. (2013). *Diagnostic and statistical Manual of Mental disorders* (5th ed.). American Psychiatric Association.

[CR2] Binde, P., Romild, U., & Volberg, R. A. (2017). Forms of gambling, gambling involvement and problem gambling: Evidence from a Swedish population survey. *International Gambling Studies*, *17*(3), 490–507. 10.1080/14459795.2017.1360928

[CR3] Brosowski, T., Hayer, T., Meyer, G., Rumpf, H. J., John, U., Bischof, A., & Meyer, C. (2015). Thresholds of probable problematic gambling involvement for the German population: Results of the pathological gambling and epidemiology (PAGE) study. *Psychology of Addictive Behaviors*, *29*(3), 794–804. 10.1037/adb0000088 26415065 10.1037/adb0000088

[CR5] Browne, M., & Rockloff, M. J. (2017). The dangers of conflating gambling-related harm with disordered gambling: Commentary on: Prevention paradox logic and problem gambling (Delfabbro & King, 2017). *Journal of Behavioral Addictions*, *6*(3), 317–320. 28889755 10.1556/2006.6.2017.059 PMC5700733

[CR4] Browne, M., Delfabbro, P., Thorne, H. B., Tulloch, C., Rockloff, M. J., Hing, N., Dowling, N. A., & Stevens, M. (2023). Unambiguous evidence that over half of gambling problems in Australia are caused by electronic gambling machines: Results from a large-scale composite population study. *Journal of Behavioral Addictions*, *12*(1), 182–193. 36729109 10.1556/2006.2022.00083 PMC10260219

[CR8] Currie, S. R., Hodgins, D. C., Wang, J., El-Guebaly, N., Wynne, H., & Chen, S. (2006). Risk of harm among gamblers in the general population as a function of level of participation in gambling activities. *Addiction*, *101*(4), 570–580. 10.1111/j.1360-0443.2006.01392.x 16548936 10.1111/j.1360-0443.2006.01392.x

[CR9] Currie, S. R., lt, Hodgins, S., Wang, J., El-Guebaly, N., H., & Wynne, H. (2008). In Pursuit of Empirically Based Responsible Gambling Limits. *International Gambling Studies*, *8*(2), 207–227. 10.1080/14459790802172265

[CR10] Currie, S. R., Miller, N., Hodgins, D. C., & Wang, J. (2009). Defining a threshold of harm from gambling for population health surveillance research. *International Gambling Studies*, *9*(1), 19–38. 10.1080/14459790802652209

[CR7] Currie, S. R., Hodgins, D. C., Casey, D. M., el-Guebaly, N., Smith, G. J., Williams, R. J., Schopflocher, D. P., & Wood, R. T. (2012). Examining the predictive validity of low‐risk gambling limits with longitudinal data. *Addiction*, *107*(2), 400–406. 10.1111/j.1360-0443.2011.03622.x 21851443 10.1111/j.1360-0443.2011.03622.x

[CR6] Currie, S. R., Hodgins, D. C., Casey, D. M., el-Guebaly, N., Smith, G. J., Williams, R. J., & Schopflocher, D. P. (2017). Deriving low-risk gambling limits from longitudinal data collected in two independent Canadian studies. *Addiction*, *112*(11), 2011–2020. 10.1111/add.13909 28623865 10.1111/add.13909

[CR11] Delfabbro, P., & King, D. L. (2019). Challenges in the Conceptualisation and Measurement of Gambling-related harm. *Journal of Gambling Studies*, *35*(3), 743–755. 10.1007/s10899-019-09844-1 30879158 10.1007/s10899-019-09844-1

[CR12] Delfabbro, P., King, D. L., Browne, M., & Dowling, N. A. (2020). Do EGMs have a Stronger Association with Problem Gambling than Racing and Casino Table games? Evidence from a decade of Australian prevalence studies. *Journal of Gambling Studies*, *36*(2), 499–511. 10.1007/s10899-020-09950-5 32306234 10.1007/s10899-020-09950-5

[CR15] Dowling, N., Smith, D., & Thomas, T. (2005). Electronic gaming machines: Are they the ‘crack-cocaine’ of gambling? *Addiction*, *100*(1), 33–45. 10.1111/j.1360-0443.2005.00962.x 15598190 10.1111/j.1360-0443.2005.00962.x

[CR13] Dowling, N. A., Greenwood, C. J., Merkouris, S. S., Youssef, G. J., Browne, M., Rockloff, M., & Myers, P. (2021a). The identification of Australian low-risk gambling limits: A comparison of gambling-related harm measures. *Journal of Behavioral Addictions*, *10*(1), 21–34. 33793416 10.1556/2006.2021.00012 PMC8969860

[CR14] Dowling, N. A., Youssef, G. J., Greenwood, C., Merkouris, S. S., Suomi, A., & Room, R. (2021b). The development of empirically derived Australian low-risk gambling limits. *Journal of Clinical Medicine*, *10*(2), 167. 33418841 10.3390/jcm10020167 PMC7824838

[CR16] Fey, C. F., Hu, T., & Delios, A. (2023). The measurement and communication of Effect sizes in Management Research. *Management and Organization Review*, *19*(1), 176–197. 10.1017/mor.2022.2

[CR17] Gambling, & Act (2018 (2018:1138)). https://rkrattsbaser.gov.se/sfst?bet=2018:1138

[CR18] Hodgins, D. C., Young, M. M., Currie, S. R., Abbott, M., Billi, R., Brunelle, N., Costes, J. M., Dufour, M., Flores-Pajot, M. C., Olason, D. T., Paradis, C., Romild, U., Salonen, A., Volberg, R., & Nadeau, L. (2023). Lower-risk gambling limits: Linked analyses across eight countries. *International Gambling Studies*, *23*(2), 328–344. 10.1080/14459795.2022.2143546

[CR19] Jonsson, J., Hodgins, D. C., Lyckberg, A., Currie, S., Young, M. M., Pallesen, S., & Carlbring, P. (2022). In search of lower risk gambling levels using behavioral data from a gambling monopolist. *Journal of Behavioral Addictions*, *11*(3), 890–899. 10.1556/2006.2022.00062 36125925 10.1556/2006.2022.00062 PMC9872526

[CR20] Korn, D. A., & Shaffer, H. J. (1999). Gambling and the Health of the Public: Adopting a Public Health Perspective. *Journal of Gambling Studies*, *15*(4), 289–365. 10.1023/A:1023005115932 12766466 10.1023/a:1023005115932

[CR21] Langeland, E., Johnsen, I. F., Sømme, K. K., Morken, A. M., Erevik, E. K., Kolberg, E., Jonsson, J., Mentzoni, R. A., & Pallesen, S. (2022). One size does not fit all. Should gambling loss limits be based on income? *Frontiers in Psychiatry*, *13*. 10.3389/fpsyt.2022.1005172 10.3389/fpsyt.2022.1005172 PMC9709812 36465287

[CR22] Langham, E., Thorne, H., Browne, M., Donaldson, P., Rose, J., & Rockloff, M. (2016). Understanding gambling related harm: A proposed definition, conceptual framework, and taxonomy of harms. *Bmc Public Health*, *16*(1), 80. 10.1186/s12889-016-2747-0 26818137 10.1186/s12889-016-2747-0 PMC4728872

[CR23] Louderback, E. R., LaPlante, D. A., Currie, S. R., & Nelson, S. E. (2021). Developing and validating lower risk online gambling thresholds with actual bettor data from a major internet gambling operator. *Psychology of Addictive Behaviors*, *35*(8), 921–938. 10.1037/adb0000628 34881914 10.1037/adb0000628

[CR24] Markham, F., Young, M., & Doran, B. (2016). The relationship between player losses and gambling- related harm: Evidence from nationally representative cross‐ sectional surveys in four countries. *Addiction*, *111*(2), 320–330. 10.1111/add.13178 26567515 10.1111/add.13178

[CR25] Molander, O., Wennberg, P., & Berman, A. H. (2023). The gambling disorders identification test (GDIT): Psychometricevaluation of a new comprehensive measure for gambling disorder and problem gambling. *Assessment*, *30*(1), 225–237. 10.1177/10731911211046045 PMC9684656 34617456

[CR26] Molander, O., Wennberg, P., Dowling, N. A., & Berman, A. H. (2024). Assessing gambling disorder using frequency- and time-based response options: A rasch analysis of the gambling disorder identification test. *International Journal of Methods in Psychiatric Research*, *33*(1), e2018. 10.1002/mpr.2018 38475935 10.1002/mpr.2018 PMC10933385

[CR40] Nagelkerke, N. J. (1991). A note on a general definition of the coefficient of determination. *biometrika*, *78*(3), 691–692.

[CR27] Quilty, L. C., Murati, A., D., & Bagby, R. M. (2014). Identifying indicators of harmful and problem gambling in a Canadian sample through receiver operating characteristic analysis. *Psychology of Addictive Behaviors*, *28*(1), 229. 23647158 10.1037/a0032801

[CR28] Room, R., & Rehm, J. (2012). Clear criteria based on absolute risk: Reforming the basis of guidelines on low-risk drinking. *Drug and Alcohol Review*, *31*(2), 135–140. 10.1111/j.1465-3362.2011.00398.x 22168584 10.1111/j.1465-3362.2011.00398.x

[CR29] Ruopp, M. D., Perkins, N. J., Whitcomb, B. W., & Schisterman, E. F. (2008). Youden Index and Optimal Cut-Point estimated from observations affected by a lower limit of detection. *Biometrical Journal*, *50*(3), 419–430. 10.1002/bimj.200710415 18435502 10.1002/bimj.200710415 PMC2515362

[CR30] Russell, A. M., Browne, M., Hing, N., Rockloff, M., Newall, P., Dowling, N. A., Merkouris, S., King, D. L., Stevens, M., & Salonen, A. H. (2023). Electronic gaming machine accessibility and gambling problems: A natural policy experiment. *Journal of Behavioral Addictions*, *12*(3), 721–732. 37594879 10.1556/2006.2023.00044 PMC10562817

[CR31] Swets, J. A., Dawes, R. M., & Monahan, J. (2000). Psychological science can improve diagnostic decisions. *Psychological Science in the Public Interest*, *1*(1), 1–26. 10.1111/1529-1006.001 26151979 10.1111/1529-1006.001

[CR32] Ukhova, D., Marionneau, V., Nikkinen, J., & Wardle, H. (2024). Public health approaches to gambling: A global review of legislative trends. *The Lancet Public Health*, *9*(1), e57–e67. 10.1016/S2468-2667(23)00221-9 37944544 10.1016/S2468-2667(23)00221-9 PMC10927617

[CR33] Walker, M., Toneatto, T., Potenza, M. N., Petry, N., Ladouceur, R., Hodgins, D. C., el-Guebaly, N., Echeburua, E., & Blaszczynski, A. (2006). A framework for reporting outcomes in problem gambling treatment research: The Banff, Alberta Consensus. *Addiction*, *101*(4), 504–511. 10.1111/j.1360-0443.2005.01341.x 16548930 10.1111/j.1360-0443.2005.01341.x

[CR34] Wall, H., Berman, A. H., Jayaram-Lindström, N., Hellner, C., & Rosendahl, I. (2021). Gambler clusters and problem gambling severity: A cluster analysis of Swedish gamblers accessing an online problem gambling screener. *Psychology of Addictive Behaviors*, *35*(1), 102. 32614206 10.1037/adb0000674

[CR39] Wall, H., Marionneau, V., Lindqvist, H., & Molander, O. (2025). Digitalisation of gambling harm? Gamblingconsumption, negative consequences, and clinical characteristics among Swedish help-seekers. *Addictive Behaviors*, *160*, 108182. 10.1016/j.addbeh.2024.108182 39353250

[CR35] Wechsler, H., Moeykens, B., Davenport, A., Castillo, S., & Hansen, J. (1995). The adverse impact of heavy episodic drinkers on other college students. *Journal of Studies on Alcohol*, *56*(6), 628–634. 10.15288/jsa.1995.56.628 8558894 10.15288/jsa.1995.56.628

[CR36] Weinstock, J., Ledgerwood, D. M., & Petry, N. M. (2007). Association between posttreatment gambling behavior and harm in pathological gamblers. *Psychology of Addictive Behaviors*, *21*(2), 185. 17563138 10.1037/0893-164X.21.2.185

[CR37] Weinstock, J., Whelan, J. P., & Meyers, A. (2008). College Students’ Gambling Behavior: When does it become Harmful? *Journal of American College Health*, *56*(5), 513–522. 10.3200/JACH.56.5.513-522 18400663 10.3200/JACH.56.5.513-522

[CR38] Youden, W. J. (1950). Index for rating diagnostic tests. *Cancer*, *3*(1), 32–35. 10.1002/1097-0142(1950)3:1%3C32::AID-CNCR2820030106%3E3.0.CO;2-3 15405679 10.1002/1097-0142(1950)3:1<32::aid-cncr2820030106>3.0.co;2-3

