# Supplementary material for: Striving Towards National Lower-Risk Gambling Guidelines: An Empirical Investigation Among a Sample of Swedish Gamblers
Source: J Gambl Stud. 2025 Jan 8;41(2):753–66. doi: 10.1007/s10899-024-10372-w (PMC12117005; doi:10.1007/s10899-024-10372-w)
Supplement: Supplementary file 1 — Supplementary Material 1 [file 10899_2024_10372_MOESM1_ESM.docx]

Supplementary table 1

Gambling frequency, lower-risk limits

|  | 1 + harms (AUC = .74) | | |  | 2 + harms (AUC = .73) | | |
| --- | --- | --- | --- | --- | --- | --- | --- |
| GDIT_item1_ | Sensitivity | Specificity | Youden's index |  | Sensitivity | Specificity | Youden's index |
| 0. Never | 1.00 | 0.00 | 0.00 |  | 1.00 | 0.00 | 0.00 |
| 1. Monthly or less | 0.88 | 0.10 | -0.02 |  | 0.86 | 0.1 | -0.04 |
| 2. 2–4 times a month | 0.84 | 0.47 | 0.31 |  | 0.85 | 0.41 | 0.26 |
| 3. 2–3 times a week | 0.71 | 0.75 | 0.45 |  | 0.74 | 0.67 | 0.41 |
| 4. 4 or more times a week | 0.54 | 0.87 | 0.40 |  | 0.63 | 0.82 | 0.45 |
| 5. Daily | 0.37 | 0.91 | 0.28 |  | 0.45 | 0.89 | 0.34 |
| 6. Several times a day | 0.17 | 0.96 | 0.13 |  | 0.22 | 0.95 | 0.17 |

Supplementary table 2

Gambling duration, lower-risk limits

| Hours gambled  past month | 1 + harms (AUC = .77) | | |  | 2 + harms (AUC = .75) | | |
| --- | --- | --- | --- | --- | --- | --- | --- |
|  | Sensitivity | Specificity | Youden's index |  | Sensitivity | Specificity | Youden's index |
| 0 | 1.00 | 0.00 | 0.00 |  | 1.00 | 0.00 | 0.00 |
| 1 | 0.86 | 0.18 | 0.04 |  | 0.85 | 0.17 | 0.02 |
| 2 | 0.85 | 0.4 | 0.25 |  | 0.84 | 0.35 | 0.2 |
| 4 | 0.82 | 0.59 | 0.41 |  | 0.83 | 0.51 | 0.34 |
| 5 | 0.81 | 0.66 | 0.47 |  | 0.82 | 0.57 | 0.39 |
| 6 | 0.77 | 0.77 | 0.55 |  | 0.80 | 0.67 | 0.47 |
| 8 | 0.76 | 0.79 | 0.55 |  | 0.79 | 0.69 | 0.48 |
| 11 | 0.75 | 0.79 | 0.54 |  | 0.79 | 0.69 | 0.48 |
| 15 | 0.69 | 0.82 | 0.51 |  | 0.75 | 0.74 | 0.49 |
| 17 | 0.64 | 0.84 | 0.48 |  | 0.72 | 0.77 | 0.49 |
| 24 | 0.61 | 0.86 | 0.46 |  | 0.68 | 0.79 | 0.48 |
| 35 | 0.53 | 0.89 | 0.42 |  | 0.6 | 0.83 | 0.43 |
| 45 | 0.48 | 0.9 | 0.39 |  | 0.57 | 0.86 | 0.43 |
| 51 | 0.47 | 0.91 | 0.38 |  | 0.55 | 0.87 | 0.42 |
| 55 | 0.45 | 0.92 | 0.37 |  | 0.52 | 0.88 | 0.4 |
| 56 | 0.39 | 0.93 | 0.32 |  | 0.45 | 0.89 | 0.34 |
| 80 | 0.35 | 0.94 | 0.29 |  | 0.42 | 0.91 | 0.33 |
| 88 | 0.3 | 0.96 | 0.26 |  | 0.36 | 0.93 | 0.29 |
| 105 | 0.25 | 0.96 | 0.21 |  | 0.32 | 0.94 | 0.26 |
| 128 | 0.24 | 0.96 | 0.2 |  | 0.31 | 0.94 | 0.25 |
| 165 | 0.19 | 0.96 | 0.15 |  | 0.25 | 0.95 | 0.2 |
| 170 | 0.18 | 0.96 | 0.14 |  | 0.23 | 0.96 | 0.18 |
| 240 | 0.14 | 0.98 | 0.12 |  | 0.18 | 0.98 | 0.16 |
| 272 | 0.10 | 0.98 | 0.09 |  | 0.14 | 0.98 | 0.12 |
| 510 | 0.05 | 0.99 | 0.05 |  | 0.07 | 0.99 | 0.06 |

Supplementary table 3

Expenditures, lower-risk cut offs

| Swedish crowns wagered,  past month | 1 + harms (AUC = .73) | | |  | 2 + harms (AUC = .67) | | |
| --- | --- | --- | --- | --- | --- | --- | --- |
|  | Sensitivity | Specificity | Youden's index |  | Sensitivity | Specificity | Youden's index |
| 0 | 0.98 | 0.02 | 0.00 |  | 0.98 | 0.02 | 0.00 |
| 10 | 0.79 | 0.29 | 0.08 |  | 0.75 | 0.26 | 0.01 |
| 14 | 0.79 | 0.29 | 0.08 |  | 0.75 | 0.26 | 0.01 |
| 20 | 0.78 | 0.29 | 0.08 |  | 0.75 | 0.26 | 0.02 |
| 25 | 0.78 | 0.3 | 0.08 |  | 0.75 | 0.27 | 0.02 |
| 30 | 0.78 | 0.3 | 0.09 |  | 0.75 | 0.27 | 0.02 |
| 40 | 0.78 | 0.31 | 0.10 |  | 0.75 | 0.28 | 0.03 |
| 50 | 0.78 | 0.32 | 0.10 |  | 0.75 | 0.28 | 0.03 |
| 90 | 0.78 | 0.33 | 0.11 |  | 0.75 | 0.30 | 0.05 |
| 100 | 0.78 | 0.33 | 0.11 |  | 0.75 | 0.30 | 0.05 |
| 110 | 0.77 | 0.41 | 0.18 |  | 0.74 | 0.36 | 0.11 |
| 120 | 0.77 | 0.42 | 0.18 |  | 0.74 | 0.37 | 0.11 |
| 125 | 0.77 | 0.42 | 0.19 |  | 0.74 | 0.37 | 0.11 |
| 130 | 0.77 | 0.42 | 0.19 |  | 0.74 | 0.37 | 0.12 |
| 150 | 0.77 | 0.43 | 0.19 |  | 0.74 | 0.37 | 0.12 |
| 175 | 0.77 | 0.45 | 0.22 |  | 0.74 | 0.39 | 0.14 |
| 200 | 0.77 | 0.45 | 0.22 |  | 0.74 | 0.39 | 0.14 |
| 250 | 0.76 | 0.52 | 0.29 |  | 0.74 | 0.46 | 0.20 |
| 297 | 0.76 | 0.54 | 0.29 |  | 0.74 | 0.47 | 0.21 |
| 300 | 0.76 | 0.54 | 0.3 |  | 0.74 | 0.47 | 0.21 |
| 350 | 0.75 | 0.58 | 0.33 |  | 0.74 | 0.50 | 0.25 |
| 400 | 0.75 | 0.58 | 0.33 |  | 0.74 | 0.51 | 0.25 |
| 450 | 0.75 | 0.6 | 0.36 |  | 0.74 | 0.52 | 0.27 |
| 500 | 0.75 | 0.61 | 0.36 |  | 0.74 | 0.53 | 0.27 |
| 600 | 0.74 | 0.69 | 0.42 |  | 0.73 | 0.60 | 0.33 |
| 700 | 0.74 | 0.71 | 0.44 |  | 0.73 | 0.61 | 0.34 |
| 800 | 0.73 | 0.71 | 0.44 |  | 0.72 | 0.62 | 0.34 |
| 900 | 0.73 | 0.72 | 0.45 |  | 0.72 | 0.63 | 0.35 |
| 1,000 | 0.73 | 0.73 | 0.45 |  | 0.72 | 0.63 | 0.35 |
| 1,200 | 0.70 | 0.77 | 0.48 |  | 0.70 | 0.67 | 0.37 |
| 1,250 | 0.70 | 0.78 | 0.48 |  | 0.70 | 0.68 | 0.38 |
| 1,500 | 0.70 | 0.78 | 0.48 |  | 0.70 | 0.68 | 0.38 |
| 1,775 | 0.69 | 0.79 | 0.48 |  | 0.70 | 0.70 | 0.39 |
| 1,850 | 0.69 | 0.79 | 0.48 |  | 0.70 | 0.70 | 0.40 |
| 2,000 | 0.69 | 0.79 | 0.48 |  | 0.70 | 0.70 | 0.40 |
| 2,500 | 0.67 | 0.83 | 0.50 |  | 0.67 | 0.73 | 0.40 |
| 3,000 | 0.66 | 0.83 | 0.49 |  | 0.67 | 0.73 | 0.39 |
| 3,050 | 0.63 | 0.86 | 0.49 |  | 0.64 | 0.76 | 0.39 |
| 3,500 | 0.63 | 0.86 | 0.49 |  | 0.64 | 0.76 | 0.39 |
| 3,600 | 0.63 | 0.86 | 0.49 |  | 0.64 | 0.76 | 0.40 |
| 4,000 | 0.63 | 0.86 | 0.49 |  | 0.64 | 0.77 | 0.40 |
| 4,100 | 0.60 | 0.87 | 0.47 |  | 0.60 | 0.78 | 0.37 |
| 5,000 | 0.59 | 0.87 | 0.47 |  | 0.59 | 0.78 | 0.37 |
| 5,500 | 0.52 | 0.9 | 0.42 |  | 0.53 | 0.82 | 0.35 |
| 6,000 | 0.52 | 0.9 | 0.42 |  | 0.53 | 0.82 | 0.35 |
| 6,500 | 0.50 | 0.90 | 0.41 |  | 0.51 | 0.82 | 0.34 |
| 7,000 | 0.50 | 0.90 | 0.40 |  | 0.51 | 0.82 | 0.33 |
| 7,500 | 0.46 | 0.91 | 0.37 |  | 0.47 | 0.84 | 0.31 |
| 8,000 | 0.46 | 0.91 | 0.37 |  | 0.47 | 0.84 | 0.30 |
| 8,500 | 0.44 | 0.91 | 0.35 |  | 0.45 | 0.85 | 0.29 |
| 10,000 | 0.43 | 0.91 | 0.35 |  | 0.44 | 0.85 | 0.29 |
| 12,000 | 0.36 | 0.93 | 0.29 |  | 0.37 | 0.88 | 0.24 |
| 13,000 | 0.34 | 0.94 | 0.28 |  | 0.36 | 0.89 | 0.25 |
| 14,000 | 0.33 | 0.94 | 0.27 |  | 0.35 | 0.89 | 0.24 |
| 14,600 | 0.33 | 0.94 | 0.27 |  | 0.35 | 0.89 | 0.24 |
| 15,000 | 0.33 | 0.94 | 0.27 |  | 0.34 | 0.89 | 0.24 |
| 16,000 | 0.27 | 0.95 | 0.22 |  | 0.29 | 0.91 | 0.20 |
| 18,000 | 0.26 | 0.95 | 0.22 |  | 0.29 | 0.92 | 0.20 |
| 19,000 | 0.25 | 0.95 | 0.21 |  | 0.28 | 0.92 | 0.19 |
| 20,000 | 0.25 | 0.95 | 0.20 |  | 0.27 | 0.92 | 0.18 |
| 24,300 | 0.19 | 0.96 | 0.15 |  | 0.19 | 0.93 | 0.12 |
| 25,000 | 0.18 | 0.96 | 0.15 |  | 0.18 | 0.93 | 0.12 |
| 26,000 | 0.17 | 0.96 | 0.14 |  | 0.17 | 0.94 | 0.11 |
| 27,000 | 0.17 | 0.96 | 0.13 |  | 0.17 | 0.94 | 0.11 |
| 30,000 | 0.16 | 0.96 | 0.13 |  | 0.17 | 0.94 | 0.10 |
| 34,000 | 0.14 | 0.97 | 0.11 |  | 0.15 | 0.95 | 0.09 |
| 35,000 | 0.14 | 0.97 | 0.11 |  | 0.15 | 0.95 | 0.10 |
| 35,500 | 0.13 | 0.97 | 0.10 |  | 0.14 | 0.95 | 0.09 |
| 37,000 | 0.13 | 0.97 | 0.10 |  | 0.14 | 0.95 | 0.09 |
| 40,000 | 0.13 | 0.97 | 0.10 |  | 0.14 | 0.95 | 0.09 |
| 42,000 | 0.12 | 0.98 | 0.10 |  | 0.12 | 0.96 | 0.08 |
| 45,000 | 0.12 | 0.98 | 0.10 |  | 0.12 | 0.96 | 0.09 |
| 50,000 | 0.11 | 0.98 | 0.09 |  | 0.12 | 0.97 | 0.08 |
| 60,000 | 0.09 | 0.98 | 0.07 |  | 0.09 | 0.97 | 0.06 |
| 70,000 | 0.08 | 0.98 | 0.07 |  | 0.09 | 0.97 | 0.06 |
| 75,000 | 0.08 | 0.98 | 0.06 |  | 0.08 | 0.97 | 0.05 |
| 80,000 | 0.07 | 0.98 | 0.06 |  | 0.08 | 0.97 | 0.05 |
| 90,000 | 0.06 | 0.99 | 0.05 |  | 0.06 | 0.98 | 0.03 |
| 100,000 | 0.06 | 0.99 | 0.05 |  | 0.06 | 0.98 | 0.04 |
| 130,000 | 0.05 | 0.99 | 0.04 |  | 0.04 | 0.98 | 0.03 |
| 133,000 | 0.04 | 0.99 | 0.03 |  | 0.04 | 0.98 | 0.02 |
| 140,000 | 0.04 | 0.99 | 0.03 |  | 0.03 | 0.98 | 0.02 |
| 143,000 | 0.04 | 0.99 | 0.03 |  | 0.03 | 0.98 | 0.01 |
| 150,000 | 0.03 | 0.99 | 0.02 |  | 0.02 | 0.98 | 0.01 |
| 200,000 | 0.03 | 1.00 | 0.02 |  | 0.02 | 0.99 | 0.01 |
| 400,000 | 0.02 | 1.00 | 0.02 |  | 0.01 | 0.99 | 0.01 |
| 450,000 | 0.02 | 1.00 | 0.02 |  | 0.01 | 0.99 | 0.00 |
| 534,125 | 0.01 | 1.00 | 0.01 |  | 0.00 | 0.99 | 0.00 |
| 850,000 | 0.01 | 1.00 | 0.01 |  | 0.00 | 1.00 | 0.00 |
| 900,000 | 0.01 | 1.00 | 0.01 |  | 0.00 | 1.00 | 0.00 |
| 1,500,000 | 0.00 | 1.00 | 0.00 |  | 0.00 | 1.00 | 0.00 |

Supplementary table 4

Expenditure as a proportion of income, lower-risk limits

| Expenditure as a proportion of income, past month | 1 + harms (AUC = .72) | | |  | 2 + harms (AUC = .67) | | |
| --- | --- | --- | --- | --- | --- | --- | --- |
|  | Sensitivity | Specificity | Youden's index |  | Sensitivity | Specificity | Youden's index |
| 0% | 0.95 | 0.04 | 0.00 |  | 0.96 | 0.04 | 0.00 |
| 1% | 0.77 | 0.38 | 0.15 |  | 0.74 | 0.33 | 0.07 |
| 2% | 0.77 | 0.52 | 0.29 |  | 0.74 | 0.44 | 0.18 |
| 3% | 0.75 | 0.58 | 0.33 |  | 0.73 | 0.50 | 0.23 |
| 4% | 0.75 | 0.66 | 0.40 |  | 0.71 | 0.56 | 0.27 |
| 5% | 0.74 | 0.69 | 0.43 |  | 0.71 | 0.58 | 0.29 |
| 6% | 0.73 | 0.71 | 0.44 |  | 0.71 | 0.62 | 0.32 |
| 7% | 0.72 | 0.73 | 0.44 |  | 0.70 | 0.63 | 0.33 |
| 8% | 0.71 | 0.74 | 0.46 |  | 0.70 | 0.64 | 0.34 |
| 9% | 0.70 | 0.76 | 0.47 |  | 0.68 | 0.66 | 0.34 |
| 10% | 0.70 | 0.78 | 0.48 |  | 0.68 | 0.67 | 0.35 |
| 11% | 0.70 | 0.78 | 0.48 |  | 0.68 | 0.68 | 0.35 |
| 12% | 0.69 | 0.79 | 0.47 |  | 0.68 | 0.69 | 0.36 |
| 13% | 0.67 | 0.80 | 0.47 |  | 0.67 | 0.70 | 0.38 |
| 14% | 0.67 | 0.81 | 0.48 |  | 0.67 | 0.71 | 0.38 |
| 15% | 0.66 | 0.82 | 0.48 |  | 0.66 | 0.72 | 0.38 |
| 16% | 0.65 | 0.83 | 0.49 |  | 0.66 | 0.73 | 0.38 |
| 17% | 0.65 | 0.83 | 0.48 |  | 0.65 | 0.73 | 0.38 |
| 18% | 0.64 | 0.85 | 0.49 |  | 0.64 | 0.74 | 0.38 |
| 19% | 0.64 | 0.86 | 0.49 |  | 0.64 | 0.75 | 0.39 |
| 20% | 0.63 | 0.86 | 0.49 |  | 0.63 | 0.75 | 0.38 |
| 21% | 0.62 | 0.86 | 0.48 |  | 0.62 | 0.76 | 0.38 |
| 22% | 0.61 | 0.86 | 0.47 |  | 0.62 | 0.76 | 0.38 |
| 23% | 0.6 | 0.86 | 0.46 |  | 0.61 | 0.77 | 0.38 |
| 24% | 0.59 | 0.86 | 0.45 |  | 0.61 | 0.77 | 0.38 |
| 25% | 0.58 | 0.86 | 0.44 |  | 0.59 | 0.78 | 0.37 |
| 26% | 0.56 | 0.87 | 0.43 |  | 0.58 | 0.78 | 0.36 |
| 27% | 0.56 | 0.87 | 0.43 |  | 0.58 | 0.79 | 0.36 |
| 28% | 0.56 | 0.87 | 0.43 |  | 0.58 | 0.79 | 0.37 |
| 29% | 0.56 | 0.87 | 0.43 |  | 0.58 | 0.79 | 0.37 |
| 30% | 0.55 | 0.87 | 0.42 |  | 0.56 | 0.79 | 0.35 |
| 31% | 0.54 | 0.88 | 0.41 |  | 0.55 | 0.8 | 0.34 |
| 32% | 0.52 | 0.88 | 0.40 |  | 0.53 | 0.8 | 0.33 |
| 33% | 0.51 | 0.88 | 0.39 |  | 0.51 | 0.81 | 0.32 |
| 34% | 0.49 | 0.89 | 0.38 |  | 0.50 | 0.81 | 0.31 |
| 35% | 0.49 | 0.89 | 0.38 |  | 0.50 | 0.81 | 0.31 |
| 36% | 0.49 | 0.89 | 0.38 |  | 0.50 | 0.81 | 0.31 |
| 38% | 0.48 | 0.89 | 0.37 |  | 0.49 | 0.81 | 0.30 |
| 40% | 0.48 | 0.89 | 0.37 |  | 0.48 | 0.82 | 0.30 |
| 41% | 0.47 | 0.89 | 0.37 |  | 0.48 | 0.82 | 0.29 |
| 42% | 0.47 | 0.89 | 0.36 |  | 0.47 | 0.82 | 0.29 |
| 43% | 0.46 | 0.90 | 0.36 |  | 0.47 | 0.82 | 0.29 |
| 44% | 0.45 | 0.90 | 0.35 |  | 0.46 | 0.83 | 0.29 |
| 45% | 0.45 | 0.90 | 0.35 |  | 0.46 | 0.83 | 0.29 |
| 46% | 0.45 | 0.90 | 0.34 |  | 0.46 | 0.83 | 0.28 |
| 47% | 0.44 | 0.90 | 0.34 |  | 0.45 | 0.83 | 0.28 |
| 48% | 0.44 | 0.90 | 0.34 |  | 0.45 | 0.83 | 0.28 |
| 49% | 0.44 | 0.90 | 0.33 |  | 0.45 | 0.83 | 0.28 |
| 50% | 0.43 | 0.90 | 0.33 |  | 0.44 | 0.83 | 0.28 |
| 52% | 0.40 | 0.90 | 0.30 |  | 0.42 | 0.84 | 0.26 |
| 53% | 0.40 | 0.90 | 0.30 |  | 0.42 | 0.85 | 0.26 |
| 54% | 0.39 | 0.90 | 0.29 |  | 0.41 | 0.85 | 0.25 |
| 55% | 0.39 | 0.90 | 0.29 |  | 0.41 | 0.85 | 0.26 |
| 56% | 0.38 | 0.90 | 0.29 |  | 0.41 | 0.85 | 0.26 |
| 57% | 0.37 | 0.90 | 0.28 |  | 0.40 | 0.86 | 0.25 |
| 60% | 0.37 | 0.90 | 0.27 |  | 0.40 | 0.86 | 0.26 |
| 61% | 0.37 | 0.91 | 0.28 |  | 0.39 | 0.87 | 0.26 |
| 62% | 0.36 | 0.91 | 0.28 |  | 0.39 | 0.87 | 0.26 |
| 65% | 0.36 | 0.92 | 0.28 |  | 0.39 | 0.87 | 0.26 |
| 67% | 0.35 | 0.92 | 0.28 |  | 0.38 | 0.87 | 0.25 |
| 68% | 0.34 | 0.93 | 0.26 |  | 0.35 | 0.88 | 0.23 |
| 69% | 0.33 | 0.93 | 0.26 |  | 0.35 | 0.88 | 0.23 |
| 70% | 0.32 | 0.93 | 0.25 |  | 0.34 | 0.88 | 0.22 |
| 71% | 0.30 | 0.93 | 0.23 |  | 0.33 | 0.89 | 0.21 |
| 72% | 0.29 | 0.93 | 0.22 |  | 0.32 | 0.89 | 0.21 |
| 75% | 0.29 | 0.93 | 0.22 |  | 0.31 | 0.89 | 0.20 |
| 76% | 0.27 | 0.93 | 0.20 |  | 0.31 | 0.90 | 0.21 |
| 77% | 0.27 | 0.93 | 0.20 |  | 0.30 | 0.90 | 0.20 |
| 79% | 0.27 | 0.93 | 0.20 |  | 0.30 | 0.90 | 0.20 |
| 80% | 0.26 | 0.93 | 0.19 |  | 0.29 | 0.90 | 0.19 |
| 81% | 0.26 | 0.93 | 0.19 |  | 0.28 | 0.90 | 0.18 |
| 83% | 0.26 | 0.93 | 0.19 |  | 0.28 | 0.90 | 0.19 |
| 84% | 0.23 | 0.93 | 0.16 |  | 0.25 | 0.91 | 0.16 |
| 86% | 0.23 | 0.93 | 0.16 |  | 0.25 | 0.91 | 0.16 |
| 87% | 0.22 | 0.93 | 0.15 |  | 0.24 | 0.91 | 0.15 |
| 88% | 0.22 | 0.93 | 0.15 |  | 0.23 | 0.91 | 0.14 |
| 90% | 0.21 | 0.93 | 0.14 |  | 0.22 | 0.91 | 0.14 |
| 91% | 0.20 | 0.93 | 0.13 |  | 0.22 | 0.92 | 0.14 |
| 93% | 0.20 | 0.94 | 0.14 |  | 0.22 | 0.92 | 0.14 |
| 100% | 0.19 | 0.94 | 0.13 |  | 0.21 | 0.92 | 0.13 |
| / / |  |  |  |  |  |  |  |

Supplementary table 5

Diversity, lower-risk limits

| Number of  problematic  gambling types | 1 + harms (AUC = .73) | | |  | 2 + harms (AUC = .71) | | |
| --- | --- | --- | --- | --- | --- | --- | --- |
|  | Sensitivity | Specificity | Youden's index |  | Sensitivity | Specificity | Youden's index |
| 0 | 1.00 | 0.00 | 0.00 |  | 1.00 | 0.00 | 0.00 |
| 1 | 0.92 | 0.49 | 0.41 |  | 0.95 | 0.42 | 0.37 |
| 2 | 0.46 | 0.79 | 0.25 |  | 0.49 | 0.75 | 0.23 |
| 3 | 0.21 | 0.91 | 0.12 |  | 0.22 | 0.89 | 0.11 |
| 4 | 0.10 | 0.96 | 0.06 |  | 0.12 | 0.96 | 0.08 |
| 5 | 0.05 | 0.98 | 0.03 |  | 0.06 | 0.98 | 0.04 |
| 6 | 0.03 | 0.98 | 0.01 |  | 0.04 | 0.98 | 0.03 |
| 7 | 0.02 | 0.99 | 0.01 |  | 0.03 | 0.99 | 0.02 |
| 8 | 0.01 | 0.99 | 0.00 |  | 0.01 | 0.99 | 0.01 |
